# Gap Junctions Contribute to the Regulation of Walking-Like Activity in the Adult Mudpuppy (*Necturus Maculatus*)

**DOI:** 10.1371/journal.pone.0152650

**Published:** 2016-03-29

**Authors:** Igor Lavrov, Lyle Fox, Jun Shen, Yingchun Han, Jianguo Cheng

**Affiliations:** 1 Institute of Fundamental Medicine and Biology, Kazan Federal University, Kazan, Russia; 2 Departments of Pain Management and Neurosciences, Anesthesiology Institute, Cleveland Clinic, Cleveland, Ohio, United States of America; 3 Department of Pathobiology, Lerner Research Institute, Cleveland Clinic, Cleveland, Ohio, United States of America; Universidade Federal do ABC, BRAZIL

## Abstract

Although gap junctions are widely expressed in the developing central nervous system, the role of electrical coupling of neurons and glial cells via gap junctions in the spinal cord in adults is largely unknown. We investigated whether gap junctions are expressed in the mature spinal cord of the mudpuppy and tested the effects of applying gap junction blocker on the walking-like activity induced by NMDA or glutamate in an *in vitro* mudpuppy preparation. We found that glial and neural cells in the mudpuppy spinal cord expressed different types of connexins that include connexin 32 (Cx32), connexin 36 (Cx36), connexin 37 (Cx37), and connexin 43 (Cx43). Application of a battery of gap junction blockers from three different structural classes (carbenexolone, flufenamic acid, and long chain alcohols) substantially and consistently altered the locomotor-like activity in a dose-dependent manner. In contrast, these blockers did not significantly change the amplitude of the dorsal root reflex, indicating that gap junction blockers did not inhibit neuronal excitability nonselectively in the spinal cord. Taken together, these results suggest that gap junctions play a significant modulatory role in the spinal neural networks responsible for the generation of walking-like activity in the adult mudpuppy.

## Introduction

Gap junctions are specific structures that link the cytoplasm of adjoining cells and enable direct electrical communications between them. Early in development, intercellular electrical coupling through gap junctions is primarily involved in neurogenesis and axonal targeting [1–3]. At later stages, electrical coupling between neurons contributes to the generation of rhythmic activities in neuronal networks [4–5]. Electrical coupling tends to synchronize spontaneous activities in different brain regions including the neocortex [6, 7], cortex [5, 8], brainstem [9], embryonic retina [10], and the spinal cord [11–12]. Although their roles in development have been extensively studied, much less is known about their function in adult spinal cord [13].

The *in vitro* forelimb-spinal cord preparation from the adult mudpuppy provides a unique opportunity to address this important question. It generates robust and stable walking-like activity that can last for several days [14–19]. The locomotor-like activity is induced by NMDA or glutamate and is manifested as alternating flexion and extension of the forelimb around the elbow joint and alternating electromyographic (EMG) bursts between the elbow flexor and extensors. The mudpuppy spinal cord may also express gap junctions as suggested by indirect anatomical and electrophysiological evidences [20, 21]. Immunohistochemistry studies suggest that gap junctions expressed in the mudpuppy retina are composed of proteins similar to those found in mammals such as mouse, rat, and human [21, 22]. Pharmacological agents that inhibit electrical coupling in mammals, also inhibit gap junction communication in the mudpuppy and other amphibians [21, 23–26].

In this study we addressed two fundamental questions. Are gap junction proteins expressed in the spinal cord of the adult mudpuppy? If they are expressed, do they contribute to the neural networks for walking? Using immunohistochemistry, we demonstrated that several connexins were expressed in the adult mudpuppy spinal cord. Using four gap junction blocks from three different classes, we demonstrated that gap junctions function to regulate the rhythmicity and amplitude of locomotor-like activity.

## Materials and Methods

### Animals

A total of 34 mudpuppies were used for the experiments: 6 for gap junction immunohistochemistry and 28 for pharmacological experiments. We only used adult animals with body lengths of 20–30 cm, which indicate the maturity of the animals [14–19]. The experimental protocols were approved by the Animal Care and Use Committee (IACUC) of the Cleveland Clinic.

### Immunohistochemistry analysis

Immunohistochemistry was performed according to manufacturer’s instructions and the protocols described [27]. Animals (n = 6) were anesthetized by bath application of tricaine methanesulfonate (MS222) (1.5 g/L) (Sigma, St. Louis, MO) and a dorsal laminectomy was performed on the first six segments of the spinal cord. Segments 1–5 of the spinal cord were removed and fixed with 4% paraformaldehyde in 0.1 M phosphate buffer (pH 7.4) for 2 hours at room temperature. The tissue samples were cryoprotected in 30% sucrose overnight at 4°C. The sections were rehydrated and then blocked with 0.3% Triton X-100 and 3% normal goat serum for 45 to 60 min. Then the slides were incubated overnight at 4°C with the primary antisera directed against mammalian connexins: Cx32 (Alpha Diagnostic, San Antonio TX, USA; Catalog # CX32A11-A), Cx36 (Invitrogen, Carlsbad, CA, USA; Catalog # 36–4600), Cx37 (Alpha Diagnostic; Catalog # CX37A11-A) and Cx43 (Alpha Diagnostic; Catalog # CX43B12-A). Then, the slides were washed with the incubation buffer and the primary antibodies were visualized by incubating for 45 min with Alexa Fluor 488 conjugated goat anti-rabbit antisera (1:1000–1500, Invitrogen, Catalog # A11008). For co-localization of connexin with the astrocyte marker glial fibrillary acidic protein (GFAP), the sections were simultaneously labeled with primary antisera directed against one of the connexins and chicken anti-GFAP (Millipore, Billerica, MA, USA, Catalog # AB5541). The anti-GFAP was visualized with Texas Red conjugated goat anti-chicken (Jackson Immunoresearch, West Grove, PA, USA, Catalog # 103-075-155). The sections were washed with PBS and coverslipped using Vectashield mounting medium (Vector Laboratories, Burlingame, CA, USA) containing DAPI and were viewed under a Leica DM5500 B upright microscope with fluorescent optics and Leica TCS-SP spectral laser scanning confocal microscope. Control sections were handled in a similar manner except that the primary antisera were not added to the incubation buffer. Images were captured using a CCD video camera and QCapture Pro imaging software from QImaging (Surrey, BC, Canada).

### In vitro preparation

The spinal cord-forelimb preparation was described elsewhere [14–19, 28–30]. Briefly, animals (n = 28) were anesthetized by bath application of MS222 (1.5 g/l). A dorsal laminectomy was performed and the first five segments of the spinal cord with part of the vertebral columns, the brachial nerves, and the forelimb were isolated from the rest of the body. The preparation was placed in a Petri dish containing 100% oxygenated Ringer’s solution (NaCl 115 mM, KCl 2 mM, CaCl_2_ 2 mM, MgCl_2_ 1.8 mM, HEPES 5 mM and glucose 1 g/l, pH 7.35). Then, the brachial plexus was exposed and the paraspinal muscles were removed. After dissection, the preparation was transferred to a dish with separate recording chambers for the forelimb and spinal cord. The vertebral column was stabilized in the dish by pinning it to the Sylgard (Dow Corning) base of the chamber. The preparation was continuously perfused with cooled (15°C) oxygenated Ringer’s solution throughout the entire experiment at a flow rate of 5 ml/min unless otherwise stated. After a recovery period of 1 hour all preparations showed a withdrawal reflex in response to pinching of the limbs with a pair of blunt forceps. Pairs of Teflon-coated silver wires (75 μm) were inserted into the elbow flexor (*Brachialis*) and extensor (*Extensor ulnae*) muscles for EMG recording. The signals were amplified, high-pass (10 Hz) and low-pass (300 Hz) filtered, monitored, and stored in a computer. Commercially available programs were used for recording and data analysis of walking-like activity (Axon Instruments/Molecular Devices, Sunnyvale, CA; Origin, Northampton, MA and SPSS, Chicago, IL).

### Neuroactive agent application

Walking-like activity was induced by continuous perfusion (20 ml/min) of D-glutamic acid (0.5 mM, Sigma, St. Louis, MO) or by NMDA (50 μM, Sigma, St. Louis, MO) with 10 μM D-serine (Sigma, St. Louis, MO) as described previously [14–15, 17–19]. Individual gap junction uncoupling agents were added to the D-glutamic acid or NMDA solutions after the walking-like activity stabilized. Dose response curves were generated for four different uncoupling agents, carbenoxolone (CBX, 0.01–2 mM), flufenamic acid (FFA, 0.01–2 mM), 1-Heptanol and 1-Octanol (0.01–3 mM) (Sigma, St. Louis, MO). Stock solutions of CBX, 1-Heptanol, and 1-Octanol were prepared in Ringer’s solution. These blockers are less toxic than other compounds and they are water soluble or form emulsions in 25% ethylene glycol reducing the need for solvents such as DMSO and ethanol that affect the excitability of spinal neurons [19, 31, 32]. FFA was dissolved in 25% polyethylene glycol (Sigma, St. Louis, MO), as it has been shown that polyethylene glycol is not neurotoxic but neuroprotective [33]. All drugs were diluted in 100–200 ml of Ringer’s solution immediately before use. Drugs were washed out after each application with Ringer’s solution for at least 30 min before the next drug application.

### Dorsal root reflex testing

The effects of the gap junction inhibitors on the excitability of spinal neurons were additionally monitored using the dorsal root reflex. The C3 dorsal root was stimulated with a 0.5 ms constant cathode current pulse using a stimulator (S88; Grass-Telefactor, Astro-Med Inc, West Warwick, RI, USA) and stimulus isolation unit (PSIU6; Grass-Telefactor, Astro-Med Inc, West Warwick, RI, USA). The stimulus intensity was gradually increased up to 1.5 times the motor threshold and the amplitude of the fast monosynaptic component of response was evaluated from EMG recordings obtained from the bipolar electrodes (A-M System, Carlsborg, WA).

### Data analysis

Measurements of the cycle frequency of EMG bursts were means of 20 cycles. EMG records were rectified and integrated for quantification as we and others have previously described [14–17, 29–30]. Integrated areas of the EMG from the flexor muscle (*Brachialis*) were averages of 10 bursts and normalized to the muscle discharge before drug application for each preparation. All data were reported as mean ± SE. Statistical significance of different groups was evaluated using one-way ANOVA with repeated measures and for non-parametric data we used Kruskal Wallis analysis. The criterion for statistical significance was set as P < 0.05 for all experiments. We used Sigmastat for statistical analysis and Sigmaplot for graph generation (Systat Software Inc).

## Results

### Immunohistochemistry of gap junctions in the adult mudpuppy spinal cord

**Fig 1:**
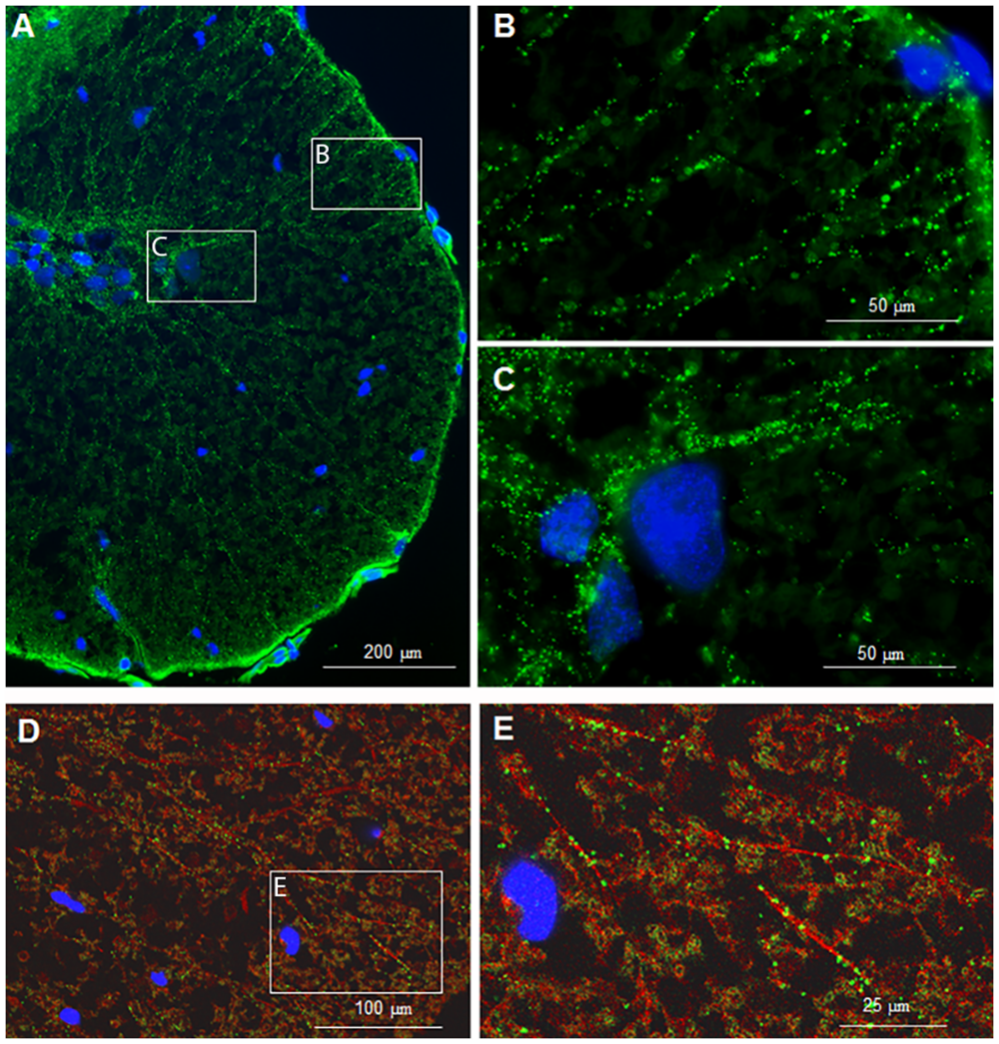
Examples of Cx43-like immunoreactivity in coronal sections of the mudpuppy spinal cord. Anti-Cx43 produced punctuate staining (green) near nuclei in the ventral horn and strings of punta radiating outward to the sub-pial plexus (A-C). Confocal images showing the colocalization of Cx43-like (green) and GFAP-like (red) immunofluorescence patterns in coronal sections of the mudpuppy spinal cord (D-E). The wide-spread distribution of the Cx43-like immunoreactivity parallels the GFAP-like staining in the gray and white matters. The confocal images are from a single optical plane. The photos on the right are higher magnification images of the regions enclosed by the white boxes. Nuclei in all images were counter stained with DAPI (blue). The scale bar in A = 200 μM; in B and C = 50 μM; in D = 100 μM; and in E = 250 μM.

Sections of the spinal cord were incubated with antisera directed against four different gap junctions’ proteins, connexin 32 (Cx32), connexin 36 (Cx36), connexin 37 (Cx37), and connexin 43 (Cx43). All four anti-connexin antisera generated punctuate labeling in the mudpuppy spinal cord and the prevalence and pattern of staining were different for each antisera. The Cx43-like staining was more abundant than the staining for the other connexins. The Cx43 antisera produced a punctuate staining on the plasma membranes of the ependymal cells surrounding the central canal (Fig 1A), punctuate staining near nuclei in the ventral horn (Fig 1C), and strings of puncta radiating outward to the sub-pial plexus (Fig 1B). Since Cx43 is predominantly expressed by astrocytes in mammals, we investigated whether Cx43 is expressed by astrocytes in the mudpuppy spinal cord by co-labeling with anti-Cx43 and antisera directed against an astrocyte marker glial fibrillary acidic protein (anti-GFAP). Images from co-labeled spinal cords demonstrated that the punctuate anti-Cx43 (green) fluorescence paralleled the anti-GFAP (red) fluorescence, a staining pattern consistent with astrocytic expression of Cx43 (Fig 1D and 1E). The Cx32 and Cx36 antibodies produced a dense punctuate staining near the nuclei of a few cells in the ventral horn and a sparse punctuate pattern in the white matter (S1 Fig). The Cx37-like immunoreactivity was rare with only a few puncta in the white matter of each section (not presented). It was not practical to determine whether the patterns of Cx32, Cx36, or Cx37 immunoreactivity co-localized with GFAP immunoreactivity because of the scarcity of Cx32, Cx36, or Cx37 staining.

### Modulation of walking-like movements and alternating flexor-extensor EMG activity by gap junction inhibitors

**Fig 2:**
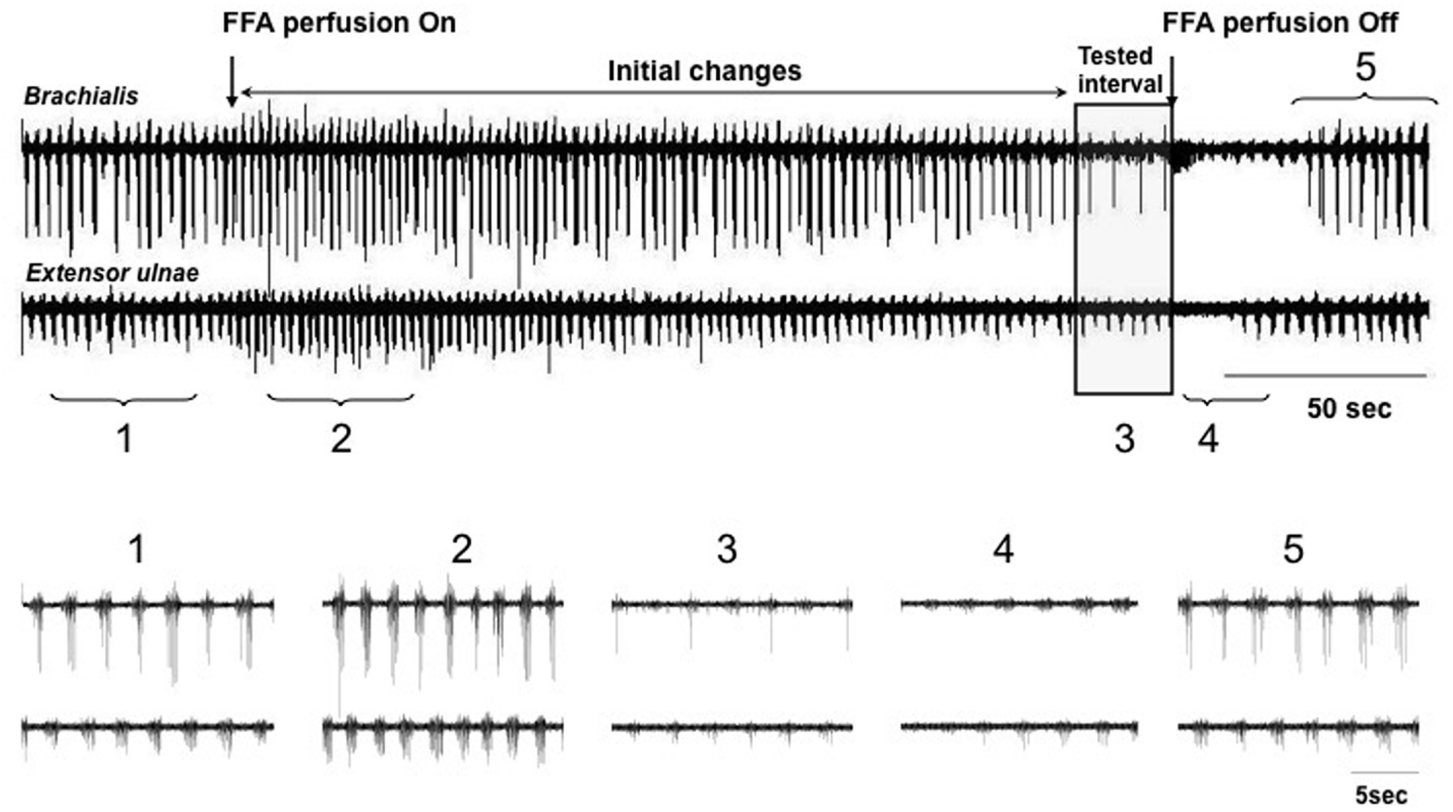
Depiction of the design for electrophysiology experiments. The figure presents the EMG activity simultaneously recorded from the flexor and extensor muscles during a typical experiment. Walking-like activity was induced with 50 μM NMDA and 10 μM D-serine. The initiation and termination of FFA application (300 μM) is indicated with arrows. Segments used for analysis of the initial changes and tested intervals are shown.

Regular rhythmic walking-like limb movement around the elbow joint and alternating flexor-extensor EMG activity were induced within the first few minutes after the application of NMDA with D-serine or D-glutamate. Fig 2 shows a representative recording of EMGs from the elbow flexor (*Brachialis*) and extensor (*Extensor ulnae*) muscles induced by NMDA (segment 1, interval before “FFA perfusion On”). The initial activity pattern consisted of alternating bursts of rhythmic activity from the flexor and extensor muscles. The average cycle frequency was 0.75 ± 0.11 Hz for D-glutamate-induced activity and 0.62 ± 0.08 Hz (n = 28; *p* < 0.05) for NMDA-induced activity. The average amplitude of the EMGs was 11 ± 3.2 mV for the *Brahialis* and 8.4 ± 2.1 mV for the *Extensor ulnae*.

The effects of the gap junction inhibitors were dose-dependent and also related to the duration of the perfusion. Fig 2 shows the effects of 0.5 mM FFA on the rhythmic activity induced by NMDA, which was continuously infused throughout the whole recording period. FFA produced an increase in cycle frequency during the first 1–2 minutes of perfusion (segment 2, “FFA perfusion On”) and decreased the frequency at later intervals (segment 3, “Tested interval”). In all cases, the walking-like activity was completely restored upon washing out of FFA with Ringer’s solution (segment 5, interval after “FFA perfusion Off”). Taking into account the time-dependent effects of the gap junction inhibitors, we analyzed the EMG activity only after the initial changes were stabilized (segment 3, “Tested interval”).

**Fig 3:**
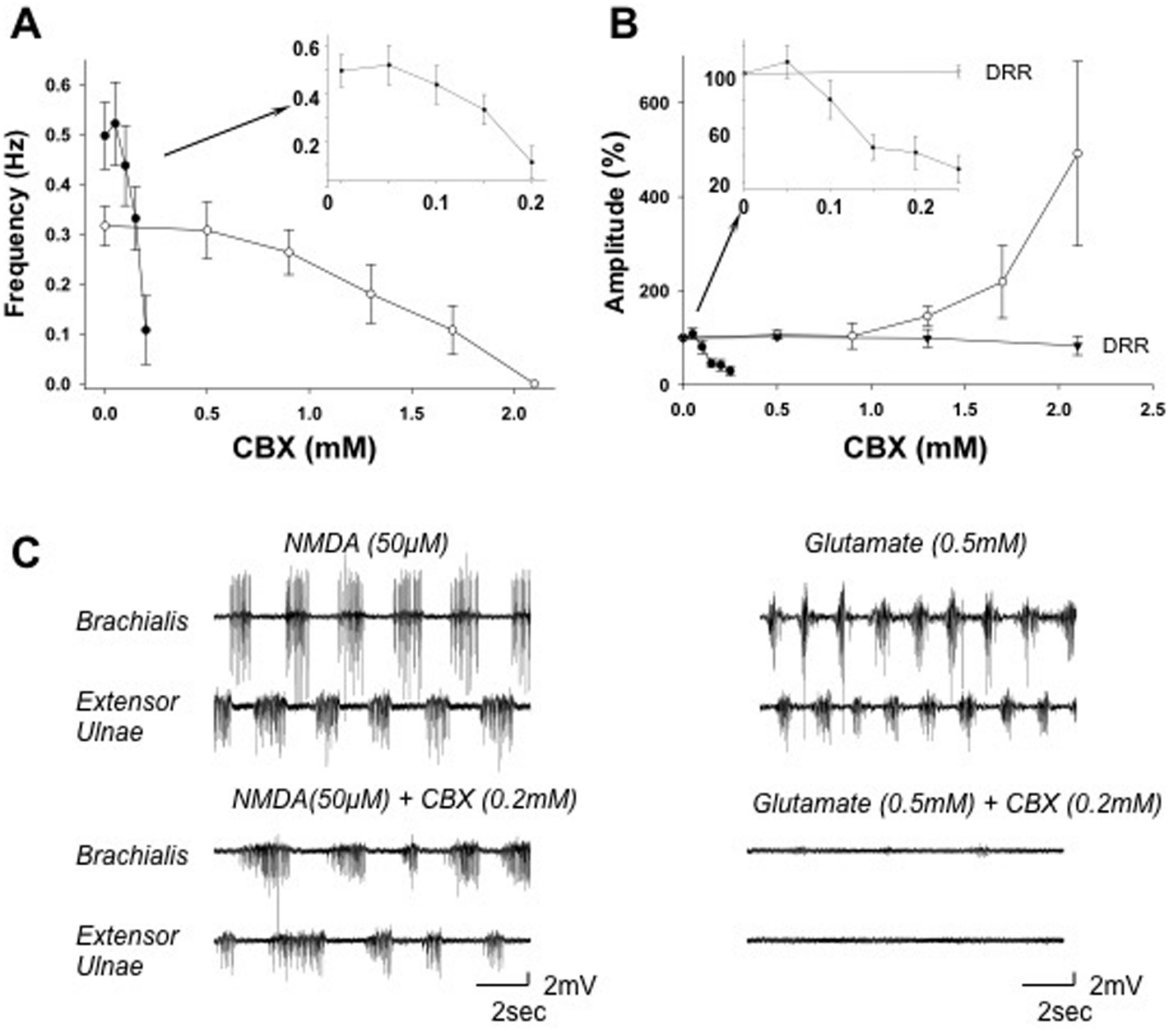
CBX modulates the NMDA- and D-glutamate-induced walking-like activity. *A*: Dose-response curves showing the effects of CBX on the cycle frequency. The mean frequency of D-glutamate- (**●**) and NMDA-induced (**○**) motor activity patterns were reduced by CBX in a dose-dependent manner. D-glutamate-induced activity was inhibited at lower concentrations than the NMDA-induced activity. *B*: Dose-response curves showing the effects of CBX on the peak EMG amplitude for D-glutamate-induced activity (**●**), NMDA-induced activity (**○**), and the dorsal root reflex (DRR, ▼). The amplitude of D-glutamate-induced activity was inhibited by CBX (concentrations > 100 μM) in a dose-dependent manner, whereas, the amplitude of NMDA-induced patterns were facilitated by CBX (concentrations > 1000 μM). In contrast, the amplitudes of the dorsal root reflexes (DRR) were not significantly affected by CBX. *C*: Paired recordings from the flexor and extensor muscles showing the effects of CBX on D-glutamate- (*right*) and NMDA-induced (*left*) motor activity patterns. Notice that the cycle frequency and EMG amplitude were reduced in the presence of 200 μM CBX (*lower pair of records*) compared to the control saline (*upper pair of records*). Values of the means and SEMs as well as their statistical significance are mentioned in the text. Data presented in all graphs are means ± SEMs.

Perfusion with low concentrations of gap junction inhibitors had a stimulatory effect on the walking-like activity, increasing the cycle frequency and/or EMG amplitude. CBX at a concentration of 50 μM increased the cycle frequency (~ 4%) and amplitude (~ 8%) of the walking-like activity induced by D-glutamate (Fig 3A and 3B). The walking-like activity induced by NMDA was less sensitive to CBX and it was not affected by CBX at concentrations < 500 μM (Fig 3). At much higher concentrations, CBX produced differential effects on the cycle frequency and EMG amplitude. The cycle frequency was reduced by CBX at concentrations > 500 μM (*p* < 0.05; *n* = 7) (Fig 3A) while the EMG amplitude was increased by CBX at concentrations >1,300 μM (*p* < 0.05; *n* = 7).

**Fig 4:**
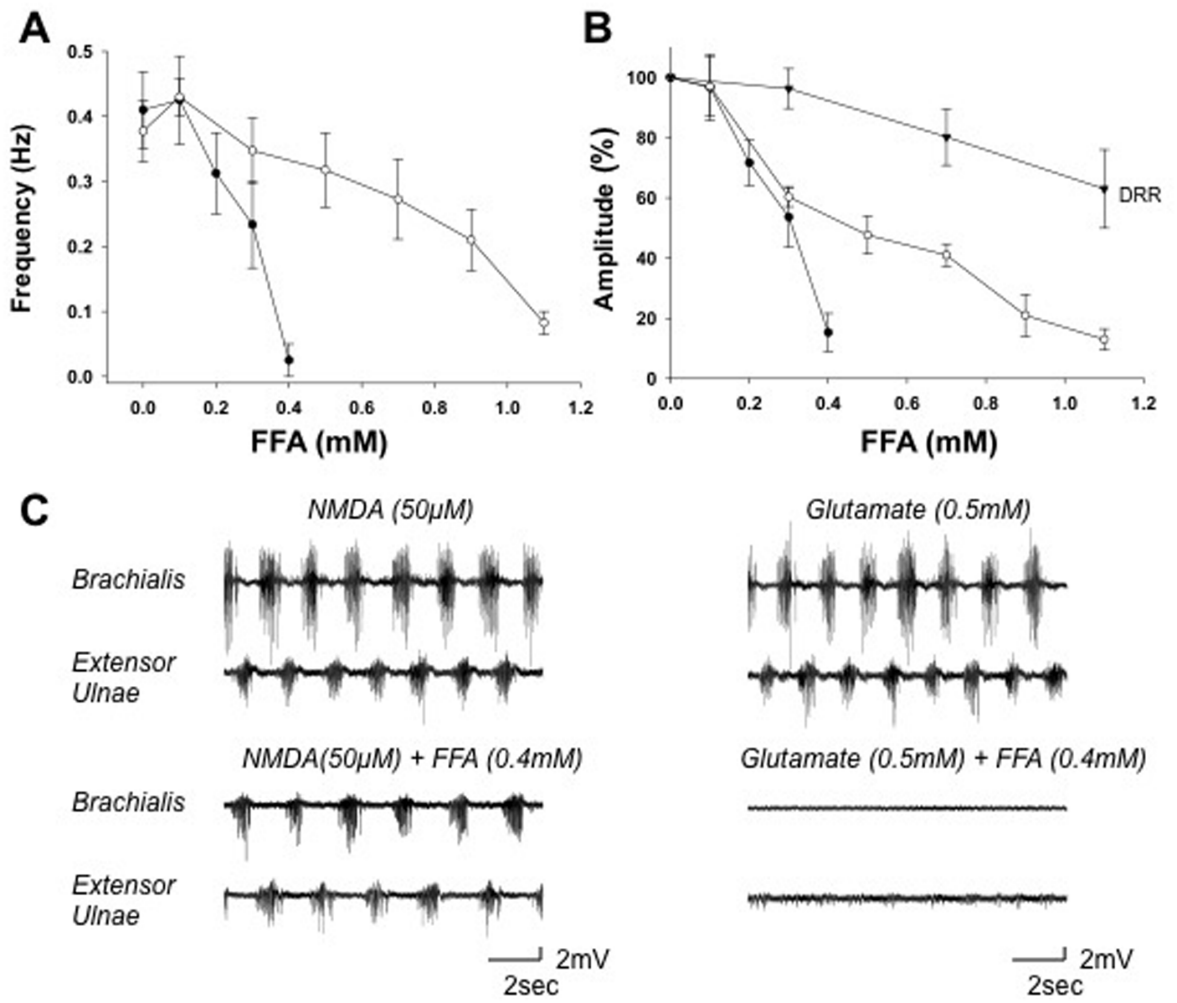
FFA modulates the NMDA- and D-glutamate-induced walking-like activity. *A*: Dose-response curves showing the effects of FFA on the cycle frequency. The mean frequency of D-glutamate- and NMDA-induced motor activity patterns were reduced by FFA concentrations > 100 μM in a dose-dependent manner. D-glutamate-induced activity was inhibited at lower concentrations than the NMDA-induced activity. *B*: Dose-response curves showing the effects of FFA on the peak EMG amplitude for D-glutamate-induced activity, NMDA-induced activity, and the dorsal root reflex. The amplitude of D-glutamate- and NMDA-induced activity was inhibited by FFA concentrations >100 μM in a dose-dependent manner. In contrast, the amplitudes of the dorsal root reflexes were not significantly affected by FFA at concentrations < 700 μM. *C*: Paired recordings from the flexor and extensor muscles showing the effects of FFA on D-glutamate- (*right*) and NMDA-induced (*left*) motor activity patterns. Notice that the cycle frequency and EMG amplitude were reduced in the presence of 500 μM FFA (*lower pair of records*) compared to the control saline (*upper pair of records*). Abbreviations, same as in Fig 3.

Application of CBX alone at a concentration that facilitated the EMGs amplitude (1,500 μM) did not induce any rhythmic activity. Instead, it prevented the initiation of walking activities by NMDA (20–100 μM), which only produced tonic activity in the presence of CBX (data not shown). The initiation of walking-like activity by D-glutamate was also completely blocked by CBX at concentrations > 300 μM (Fig 3A and 3B). In contrast, the amplitude of the dorsal root reflex was not significantly affected (*p* > 0.05; *n* = 7) (Figs 3B and 4B), indicating that the excitability of motoneurons were not inhibited nonspecifically [34–39]. This is consistent with the observation that CBX increased the EMG amplitude. These data suggest that electrical coupling through gap junctions is required to generate walking-like activity by glutamate or NMDA.

Low concentrations of other gap junction inhibitors also caused a transient increase in the cycle frequency of the walking-like activity induced by D-glutamate, or to a less extent, by NMDA. After 3 min of perfusion with 100 μM FFA, the mean cycle frequency of the NMDA-induced activity increased by 16 ± 6% (*p* < 0.05; *n* = 7) (Fig 4A). The amplitude of the EMG bursts was not facilitated by at this concentration (Fig 4B). At concentrations > 300 μM, FFA produced a dose-dependent inhibition of both cycle frequency and EMG burst amplitude. It completely blocked the activity induced by D-glutamate at 400–500 μM and the activity induced by NMDA at 1200–1300 μM (Fig 4A and 4B). The effects of FFA at 400 μM on activities induced by NMDA and glutamate are shown in Fig 4C.

**Fig 5:**
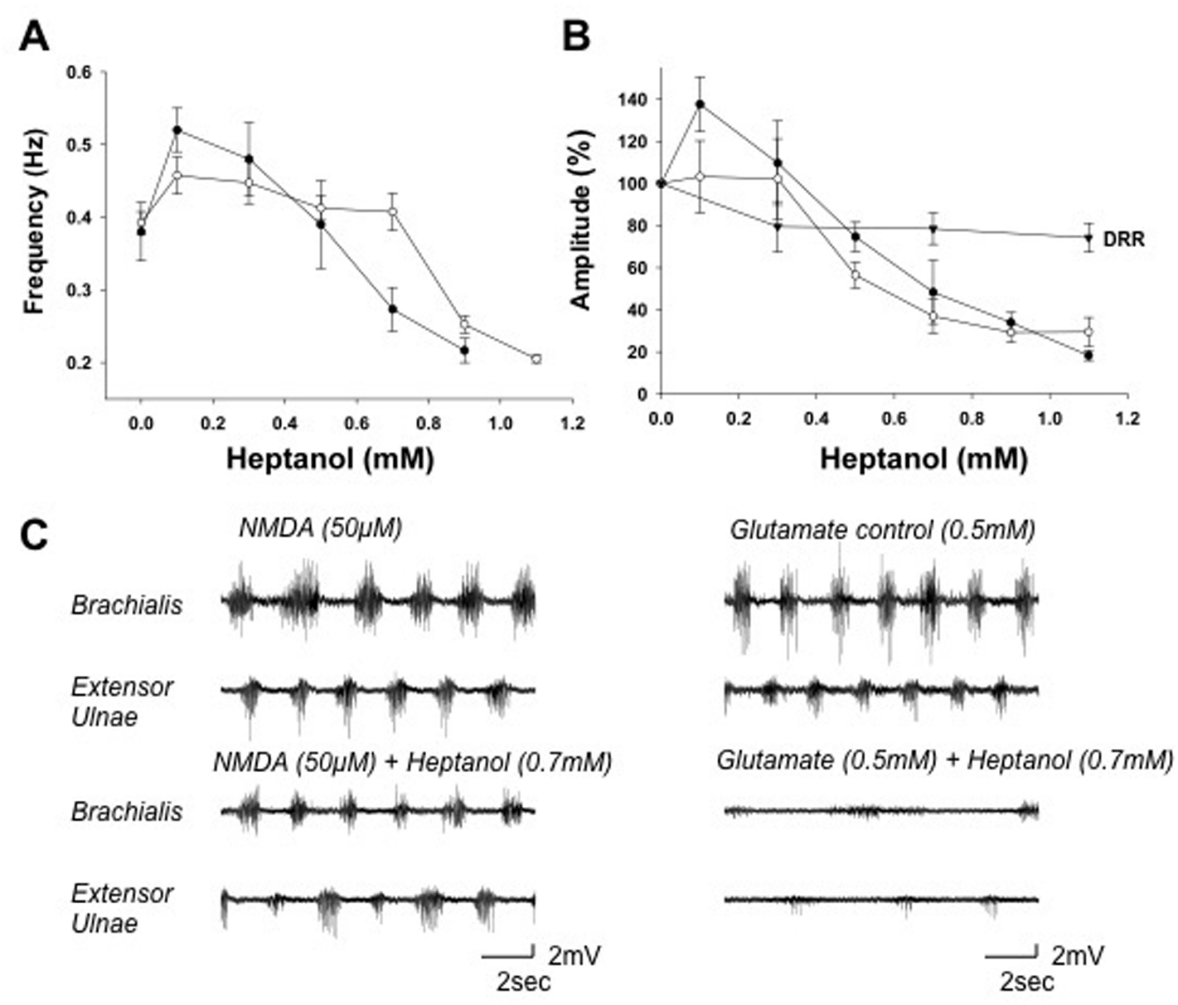
Heptanol modulates the NMDA- and D-glutamate-induced walking-like activity. *A*: Dose-response curves showing the effects of Heptanol on the cycle frequency. The mean frequency of D-glutamate- and NMDA-induced (**○**) motor activity patterns was facilitated by Heptanol at low concentrations (100–300 μM) and reduced at concentrations >400 μM in a dose-dependent manner. D-glutamate-induced activity was inhibited at lower concentrations than the NMDA-induced activity. *B*: Dose-response curves showing the effects of Heptanol on the peak EMG amplitude for D-glutamate-induced activity, NMDA-induced activity, and the dorsal root reflex. The amplitude of D-glutamate- and NMDA-induced activity was facilitated by Heptanol at low concentrations (100–300 μM) and depressed at concentrations >400 100–300 μM. The amplitudes of the dorsal root reflexes were reduced (~20%) by Heptanol at 300 μM. *C*: Paired recordings from the flexor and extensor muscles showing the effects of Heptanol on D-glutamate- (*right*) and NMDA-induced (*left*) motor activity patterns. Notice that the cycle frequency and EMG amplitude were reduced in the presence of 700 μM Heptanol (*lower pair of records*) compared to the control saline (*upper pair of records*). Abbreviations, same as in Fig 3.

**Fig 6:**
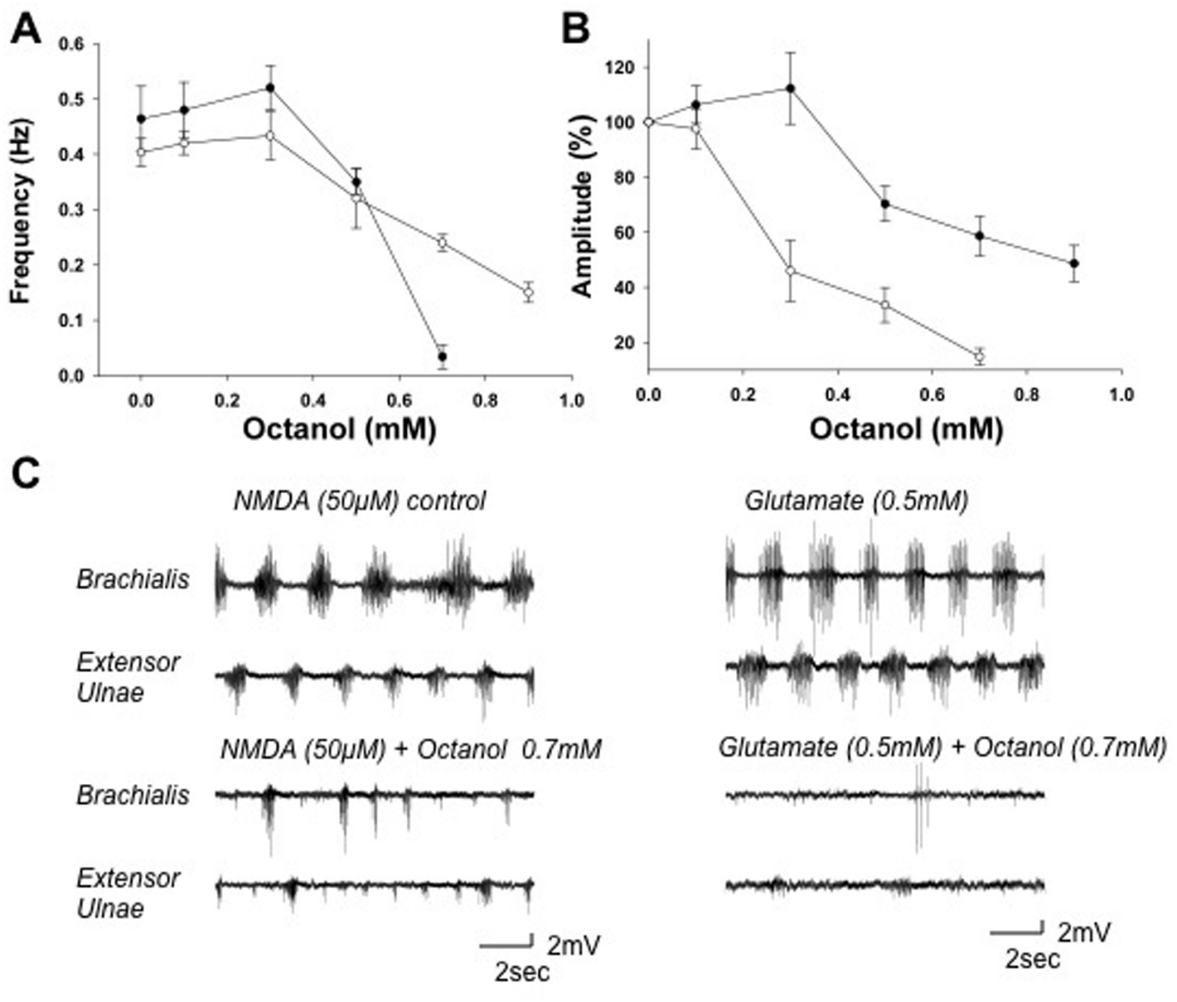
Octanol modulates the NMDA- and D-glutamate-induced walking-like activity. *A*: Dose-response curves showing the effects of Octanol on the cycle frequency. The mean frequency of D-glutamate- and NMDA-induced motor activity patterns were reduced by Octanol concentrations >400 μM in a dose-dependent manner. *B*: Dose-response curves showing the effects of Octanol on the peak EMG amplitude for D-glutamate- and NMDA-induced activity. The amplitude of D-glutamate- and NMDA-induced activity was inhibited by Octanol concentrations >400 μM in a dose-dependent manner. *C*: Paired recordings from the flexor and extensor muscles showing the effects of Octanol on D-glutamate- (*right*) and NMDA-induced (*left*) motor activity patterns. Notice that the cycle frequency and EMG amplitude were reduced in the presence of 700 μM Octanol (*lower pair of records*) compared to the control saline (*upper pair of records*). Abbreviations, same as in Fig 3.

Similarly, Heptanol at 100 μM increased the cycle frequency by 15 ± 6% in NMDA-induced activity and 36 ± 4% in D-glutamate-induced activity (*p* < 0.05; *n* = 7) (Fig 5A). Both the cycle frequency and burst amplitude of the activity induced by glutamate were inhibited by Heptanol at concentrations > 600 μM, whereas those induced by NMDA were inhibited at concentrations > 800 μM (*p* < 0.05; *n* = 7) (Fig 5). Octanol at 300 μM increased the burst amplitude by 13 ± 7% (*p* < 0.05; *n* = 7) but did not affect the cycle frequency of the walking-like activity induced by NMDA (Fig 6A). At concentrations >400 μM, Octanol inhibited the locomotor-like activities induced by either glutamate or NMDA (*p* < 0.05; *n* = 7) (Fig 6). These long chain alcohols reduced the dorsal root reflex by ~20%, as exemplified in Fig 5B, but inhibited the locomotor-like activity to a much greater extent.

The walking-like activity induced by D-glutamate was more sensitive to gap junction inhibitors than that induced by NMDA (Table 1). The half maximal inhibitory concentration (IC_50_) of CBX on the cycle frequency of glutamate-induced activity was 150 μM and it was ~ 10 fold higher for the NMDA-induced walking activity (Fig 3A). The IC_50_ of FFA was 300 μM for glutamate-induced activity and 900 μM for NMDA-induced activity (Fig 4A). The IC_50_ of Heptanol was 900 and 1100 μM (Fig 5A) and the IC_50_ of Octanol was 600 and 800 μM respectively for the glutamate- and NMDA-induced walking activities (Fig 6A).

**Table 1:** The half maximal inhibitory concentration (IC_50_) of gap junction blockers on the locomotor-like activities.

	IC_50_ (mM)	
	D-glutamate-induced activity	NMDA-induced activity
CBX	0.15	~1.5
FFA	0.3	0.9
Heptanol	0.9	1.1
Octanol	0.6	0.8

## Discussion

Gap junctions consist of six protein subunits (connexins, Cxs) [40] and in vertebrates, they are found in almost all tissues. They are regulated *in vivo* by a number of factors including pH, voltage, calcium levels, and phosphorylation [41–43]. Their expression in the mammalian spinal cord was originally described as transient with ubiquitous presence in immature animals and less common or even absent in adults [44–47]. Anatomical and physiological studies indicate that gap junctions may serve important physiological functions in the brain and spinal cord in the mammalian [3, 48–49] and vertebrate animals such as the frog [47, 50], the lamprey [51], and the goldfish [52]. However, they may play different roles in the developing and the mature stages of the central nervous system. For example, in the mammalian respiratory system, gap junction blockers cause a dose-dependent inhibition of rhythmic activity in neonatal animals, but an increase of rhythmic activity in adults [53, 54].

Our data indicate that Cx43 is the most abundant gap junction protein in the adult mudpuppy spinal cord, both in the white and gray matters (Fig 1). This is consistent with findings in the rat [55]. Cx43 has been shown to form channels between astrocytes and astrocytes and oligodendrocytes with immunoreactivity primarily observed in astrocytes and rarely in microglia. The ubiquity of Cx43 allows astrocytes to form a functional syncytium that links various regions of the CNS parenchyma in terms of flow of current, ions, and small metabolites [56]. This system also acts as a K^+^ sink following intense neuronal activity [57] and allows propagation of Ca2^+^ waves [58–60]. Our data raised the possibility that Cx43 and astrocytes may be involved in the regulation of locomotion.

We found that Cx36 was expressed sparsely in the cell body and the processes of neurons in the spinal cord. A previous study has shown that Cx36 was strongly expressed in immature neurons in rats [61]. It is possible that the expression has been reduced during development and the remaining Cx36 in adulthood may play a role in the modulation of locomotor activity by synchronizing neuronal activities. We observed positive staining for Cx32 in areas surrounding neurons. This is consistent with the finding that Cx32 was expressed predominantly in oligodendrocytes throughout adulthood [61]. In contrast, the staining for Cx37 was sparse in the mudpuppy spinal cord, consistent with the finding that Cx37 is only abundant in embryonic tissues and is expressed in the endothelium of large blood vessels, but not in capillaries, in the brain and other tissues [62]. In summary, the immunohistochemistry data provide evidence that specific gap junction proteins are expressed in the adult mudpuppy spinal cord and may be involved in the generation or modulation of locomotor activities.

Using a battery of gap junction inhibitors, we demonstrated a critical role the gap junction proteins may play in the neural networks for locomotion. It is clear that at high concentrations, all gap junction inhibitors completely blocked the ongoing walking-like activity induced by NMDA or glutamate. In addition, the gap junction inhibitors also prevented NMDA or glutamate from inducing walking-like activities. These effects are not caused by nonspecific inactivation of motoneurons by the gap junction inhibitors, because in the presence of high concentrations of gap junction inhibitors, tonic motor output was consistently induced by NMDA or glutamate, the dorsal root reflex was reliably evoked, and the walking-like activity was completely reversed after washing out the inhibitors.

The main nonspecific cytotoxic effect of CBX was described for concentrations >100μM [36, 63]. CBX, FFA, and the alcohols may also alter the function of ion channels, reduce neuronal excitability [34–39], and inhibit excitatory synaptic transmission [64–66]. Given these considerations, we started with concentrations that were previously shown to block gap junctions with minimal side-effects [67–72] and limited the duration of exposure. The resumption of the locomotor-like activity upon washing out the inhibitors in all cases and the readily evoked dorsal root reflex suggest that the excitability of the spinal neurons was not significantly affected. Octanol and Heptanol induced fast inhibition of the walking-like pattern, compared to other inhibitors. This may be related to its effect on T-type calcium channels that contribute to generation of locomotor activity in lamprey [73, 74]. Collectively, these results suggest that gap junctions play a vital role in the neural networks that are responsible for the generation of locomotion.

We further showed that the effects of the gap junction inhibitors are complex. They are excitatory at low concentrations and inhibitory at high concentrations. At low concentrations the inhibitors increased the cycle frequency and/or EMG amplitude. At high concentrations or after prolonged exposure, the gap junction inhibitors reduced the cycle frequency and/or EMG amplitude or blocked the walking-like activity altogether. Studies of the respiratory rhythm in adult rodent preparations have shown that the inhibition of gap junctions significantly increases the cycle frequency and reduces the amplitude of the phrenic nerve discharges [46]. In contrast, studies on neonatal or postnatal mice have reported a decrease in the cycle frequency without any change in the amplitude of the nerve discharge [36]. The different effects of the gap junction inhibitors in these studies may reflect functional changes of gap junctions in developmental stages from the neonate to the mature nervous systems and/or methodological differences [46].

Although there were differences in the extent, to which various gap junction inhibitors modulate the walking-like activity, the main effects were consistent across inhibitors and reproducible on the walking-like activity induced either by NMDA or by glutamate. The IC_50_ of each inhibitor is a measure of its efficacy/potency in blocking the ongoing function of the gap junction proteins. We found that the IC_50_ of each gap junction inhibitor on NMDA-induced activity differed from that on the glutamate-induced activity. This difference likely reflects the susceptibility of the intercellular connections in the neural networks for walking that are activated by NMDA and glutamate respectively. The glutamate-induced locomotor-like activity was more sensitive to gap junction inhibitors, suggesting that electrical coupling plays a more significant physiological role in generating walking-like activity by neural networks that are activated by the naturally existing neurotransmitter glutamate. It is worth noting that the mechanisms of NMDA and glutamate induced locomotor-like activities may differ. In contrast to NMDA, which is a synthetic compound that induces locomotor-like activity through activating NMDA receptors, glutamate is a naturally existing neurotransmitter that may induce rhythmic activity through NMDA, non-NMDA, and metabotropic glutamate receptors [18, 75, 76].

A significant limitation of this study is that we were unable to differentiate the roles played by each individual connexins. None of the available gap junction inhibitors are specific enough to allow us achieve this objective. Some blockers, such as the hemichannel inhibitors lanthanum chloride and mefloquine, were described to be more selective to specific gap junctions. However, recent studies indicate that most of these inhibitors are not specific, as extracellular glutathione accumulation can be equally blocked by lanthanum chloride as well as by carbenoxolone and flufenamic acid [77]. The effects of these blockers become less specific and they may affect most of the other gap junctions in higher concentrations. For example, at low concentrations, mefloquine (1 μM) blocks Cx36 and Cx50 channels expressed in neuroblastoma cells, but it inhibits Cx26, Cx32, and Cx43 channels at higher concentrations (30 μM) [78]. In both vertebrates and invertebrates, gap junctions typically couple similar types of interneurons and motoneurons with restricted distributions in the central nervous system [7, 25, 26, 36]. More than 20 different connexin genes have been identified with similar, but not identical, pharmacological properties [79–81]. A better understanding of the connexin proteins will help elucidate their specific roles in the generation and regulation of locomotor function.

Taken together, our immunohistochemistry data provided evidence for the expression of specific gap junction proteins in the adult mudpuppy spinal cord. Our pharmacological intervention experiments further demonstrated a vital role of gap junctions in the modulation of the walking-like activity. Multiple gap junction inhibitors were able to modulate the cycle frequency and EMG amplitude in a fairly consistent manner. These data support the notion that intercellular coupling through gap junctions plays a critical role in the control of locomotion in adult animals.

## Conflict of Interest/Disclosures

None of the authors, Igor Lavrov, Lyle E. Fox, Jun Shen, Yingchun Han, or Jianguo Cheng, or their immediate families have any actual or potential commercial associations that might create a conflict of interest in connection with this manuscript.

## Supporting Information

S1 Fig Examples of Cx32 (A and B) and Cx36-like (C) immunoreactivity in coronal sections of the mudpuppy spinal cord. The Cx32 and Cx36 antibodies produced a dense punctuate staining near the nuclei of a few cells in the ventral horn (A and C) and a sparse punctuate pattern in the white matter (B). Nuclei in the images were counter stained with DAPI (blue). The scale bar = 50 μM for Cx36. The scale bar = 40 μM for Cx32.
(TIF)
