# Supplementary figures and images for: Gap Junctions Contribute to the Regulation of Walking-Like Activity in the Adult Mudpuppy (Necturus Maculatus)
Source: PLoS One. 2016 Mar 29;11(3):e0152650. doi: 10.1371/journal.pone.0152650 (PMC4811563; doi:10.1371/journal.pone.0152650)

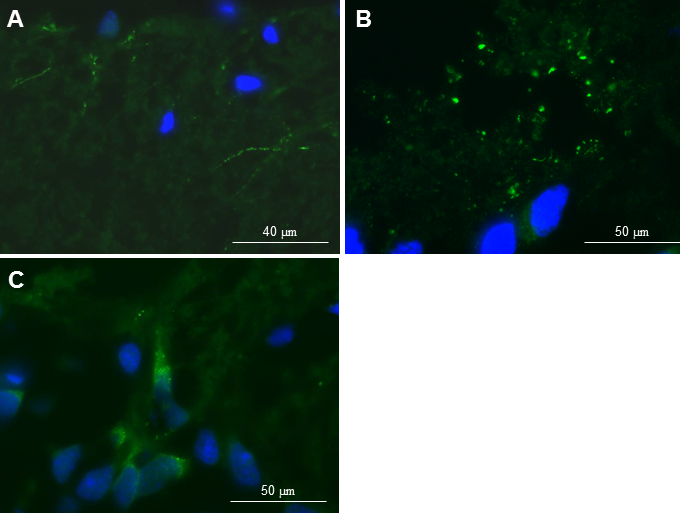

Supplement: S1 Fig — The Cx32 and Cx36 antibodies produced a dense punctuate staining near the nuclei of a few cells in the ventral horn (A and C) and a sparse punctuate pattern in the white matter (B). Nuclei in the images were counter stained with DAPI (blue). The scale bar = 50 μM for Cx36. The scale bar = 40 μM for Cx32. (TIF) [file pone.0152650.s001.tif]
